# Who is at Risk of Parkinson Disease? Refining the Preclinical Phase of *GBA1* and *LRRK2* Variant Carriers: a Clinical, Biochemical, and Imaging Approach

**DOI:** 10.1007/s11910-023-01259-1

**Published:** 2023-03-07

**Authors:** Elisa Menozzi, Anthony H. V. Schapira, Fabio Blandini, Micol Avenali

**Affiliations:** 1 Department of Clinical and Movement Neurosciences, UCL Queen Square Institute of Neurology, London, UK; 2 Aligning Science Across Parkinson’s (ASAP) Collaborative Research Network, Chevy Chase, Montgomery County, MD 20815 USA; 3 Department of Brain and Behavioral Sciences, University of Pavia, Pavia, Italy; 4 Ca’Granda IRCCS Foundation-Ospedale Maggiore Policlinico, Milan, Italy; 5 IRCCS Mondino Foundation, Neurorehabilitation Unit and Movement Disorder Center, Pavia, Italy

**Keywords:** Parkinson disease, GBA1, Glucocerebrosidase, LRRK2, Carrier, Prodromal

## Abstract

**Purpose of Review:**

Genetic variants in *GBA1* and *LRRK2* genes are the commonest genetic risk factor for Parkinson disease (PD); however, the preclinical profile of *GBA1* and *LRRK2* variant carriers who will develop PD is unclear. This review aims to highlight the more sensitive markers that can stratify PD risk in non-manifesting *GBA1* and *LRRK2* variant carriers.

**Recent Findings:**

Several case–control and a few longitudinal studies evaluated clinical, biochemical, and neuroimaging markers within cohorts of non-manifesting carriers of *GBA1* and *LRRK2* variants.

**Summary:**

Despite similar levels of penetrance of PD in *GBA1* and *LRRK2* variant carriers (10–30%), these individuals have distinct preclinical profiles. *GBA1* variant carriers at higher risk of PD can present with prodromal symptoms suggestive of PD (hyposmia), display increased α-synuclein levels in peripheral blood mononuclear cells, and show dopamine transporter abnormalities. *LRRK2* variant carriers at higher risk of PD might show subtle motor abnormalities, but no prodromal symptoms, higher exposure to some environmental factors (non-steroid anti-inflammatory drugs), and peripheral inflammatory profile. This information will help clinicians tailor appropriate screening tests and counseling and facilitate researchers in the development of predictive markers, disease-modifying treatments, and selection of healthy individuals who might benefit from preventive interventions.

## Introduction

In the last 20 years, we have witnessed an exponential and relentless growth of Parkinson disease (PD) cases, with more than 6 million people currently affected worldwide. Based on population projections, it is estimated that this number will double by 2040 [1]. The negative impact that the global burden of PD will have on those individuals primarily affected (patients and caregivers), economics and societies, is substantial [2]. Research efforts should therefore focus on interventions designed to prevent disease occurrence in healthy individuals and slow down progression in the early stages of manifest disease [3].

Individuals with a genetic predisposition for PD could be the first to be directed towards these treatments. Variants in the glucocerebrosidase (*GBA1*) gene, causing the lysosomal storage disorder Gaucher disease (GD) when present on both alleles, and leucine-rich repeat kinase 2 (*LRRK2*) gene are found in 10–15% and 1–2% of sporadic PD cases, respectively [4, 5], constituting the most important genetic risk factor for PD. The frequency of *GBA1* variants and *LRRK2* G2019S variant—the commonest—is even higher in specific populations. For instance, *GBA1* variants and *LRRK2*-G2019S are found in up to 30% of individuals of Ashkenazi Jewish ancestry and north African Arabs, respectively [4, 6]. On the contrary, *LRRK2*-G2019S is found in only 0.1% of individuals of Asian background [4]. As a whole, PD patients carrying *GBA1* variants are characterized by a more severe clinical phenotype compared with patients negative for variants in *GBA1* and *LRRK2* (hereinafter, referred to as sporadic PD, sPD) [7, 8]. *GBA1*-PD patients tend to present symptoms at earlier age and develop more severe non-motor symptoms (olfactory dysfunction, dysautonomia, cognitive decline, and psychiatric symptoms), particularly those carrying complex or severe pathogenic variants as compared to those carrying mild pathogenic or risk variants (the latter increasing PD risk but not being pathogenic for GD), supporting a genotype–phenotype association [8, 9]. Conversely, PD patients carrying *LRRK2* variants tend to show a milder phenotype and a more benign disease course [4, 10, 11]. A possible genotype–phenotype association has been observed also for *LRRK2*, with pathogenic variants being associated with a milder disease compared with risk variants [12]. Several pathogenic mechanisms linked to *GBA1* and *LRRK2* variants might be responsible for the neurodegenerative changes seen in PD. Among others, reduced enzymatic activity of glucocerebrosidase and its complex interplay with α-synuclein pathology, alterations in the autophagy-lysosomal pathway (ALP), unfolded protein response (UPR), and endoplasmic reticulum (ER) stress have been implicated in *GBA1*-PD pathogenesis [12]. More recently, lipid dyshomeostasis has been linked to specific *GBA1* risk variants [13]. Alterations in ALP, ER stress, and UPR have also been proposed as pathogenic mechanisms linked to *LRRK2* variants [12].

Despite the increasing knowledge surrounding *GBA1*- and *LRRK2*-PD, a main question related to the penetrance of these variants remains unsolved. In fact, penetrance of *GBA1* and *LRRK2* variants in PD is low and incomplete (10–30%) [4, 14], meaning that only a minority of carriers will develop the condition over their lifetime. Investigating which factors can modulate PD risk in these individuals is, therefore, imperative to (1) provide the more appropriate genetic counseling and (2) address only the high-risk individuals to preventive treatments.

Herein, we present and critically discuss the more recent evidence related to preclinical/prodromal markers associated with increased risk of PD, classified as clinical, genetic, environmental, biochemical, and neuroimaging, in *GBA1* and *LRRK2* non-manifesting carriers (NMC). The purpose is to highlight information that can help guide the design of future research studies assessing cohorts of *GBA1*- and *LRRK2*-NMC.

## Clinical Markers

### Non-motor Symptoms

Prodromal non-motor symptoms may help determine PD risk. Current evidence suggests that certain non-motor symptoms, particularly if clustered together, could represent predictive factors for PD in *GBA1*-NMC but not in *LRRK2*-NMC.

#### REM Sleep Behavior Disorder (RBD).

RBD remains one of the strongest prognostic factors for PD conversion. A recent meta-analysis concluded that *GBA1*-PD patients carrying pathogenic variants display higher risk of RBD and higher RBD Screening Questionnaire (RBDSQ) scores, whereas *LRRK2*-PD patients show no difference or reduced risk compared with sPD [15]. This could suggest an increased prevalence of RBD within *GBA1*-NMC carrying pathogenic variants; however, current evidence is conflicting. Pooled analysis of cross-sectional studies showed similar rates of RBD between *GBA1*-NMC and healthy individuals who were negative for *GBA1* or *LRRK2* variants (hereinafter, referred to as healthy controls, HC) [15]. Nevertheless, a few longitudinal studies conducted on a group of *GBA1*-NMC based in the UK showed deterioration in RBDSQ scores compared with HC over time, despite similar values at baseline [16•, 17]. *GBA1* variants were also significantly more common (9.5%) in patients with a diagnosis of idiopathic RBD (iRBD) made by video-polysomnography (PSG), compared with controls (4.1%), and *GBA1*-positive iRBD patients showed significantly increased rate of conversion to neurodegenerative conditions (mainly PD) compared with *GBA1*-negative iRBD [18]. No association was found between *LRRK2* variants and iRBD [19].

Screening for RBD by PSG is superior in terms of diagnostic accuracy than clinical questionnaires [20] and should therefore be considered in *GBA1*-NMC. The recently proposed classification of PD subtypes into “body-first” and “brain-first” suggests that premotor RBD represents a highly predictive marker only of the body-first subtype [21]. Whether and how this classification applies to genetically stratified cohorts, such as *GBA1*-NMC, is an intriguing question for future studies.

#### Hyposmia and Cognition.

Several longitudinal studies have consistently reported that olfactory function deteriorates more rapidly in *GBA1*-NMC and GD patients—carrying biallelic pathogenic variants in *GBA1*—compared with HC [16•, 22, 23]. Reduced olfactory function was also found to correlate with worse motor outcome over time and was associated with poorer cognition scores [24] and was the only premotor sign showed by one GD patient of the UK cohort, who later developed PD [16•]. However, in case–control studies conducted on large cohorts of *GBA1*-NMC, no significant differences in olfactory function between *GBA1*-NMC, *LRRK2*-NMC, and HC were found [24–26].

Similar results have been obtained for cognitive function. Large case–control studies failed to identify significant differences across groups (*GBA1*-NMC, *LRRK2*-NMC, and HC) [25–27]. However, a trend towards lower Montreal Cognitive Assessment scores in *GBA1*-NMC compared with HC, *LRRK2*-NMC, and double mutant *LRRK2*-*GBA1*-NMC [28] or subtle alterations in specific cognitive domains, such as verbal memory or executive functioning, in *GBA1*-NMC [29], were detected in a few studies.

Overall, hyposmia or cognitive deficits may not be present in most *GBA1*-NMC. However, in a subset, subtle deficits in these domains, especially if clustered together [23], can represent an enhanced risk for PD conversion.

#### Other Non-motor Symptoms.

Psychiatric features and mood disorders have been reported at increased frequency in *GBA1*-PD compared to sPD [8]. In cross-sectional studies, increased rate of apathy and anxiety was reported in *GBA1*-NMC vs. HC, but not in *LRRK2*-NMC [30]. Longitudinal studies also reported deterioration in mood in *GBA1*-NMC and GD patients, which was found to be correlated with worse olfactory function at baseline [16•].

Data regarding incidence of other prodromal symptoms, such as constipation, in *GBA1*- or *LRRK2*-NMC is still lacking. A few studies detected constipation in cohorts of GD patients [31] but the significance of these results is unclear.

### Motor Profile

As opposed to non-motor symptoms, current evidence suggests that motor function could be an important predictive factor of PD conversion in *LRRK2*-NMC, especially those individuals carrying the *LRRK2*-G2019S variant—the most studied population. Many studies have, in fact, consistently reported higher MDS-UPDRS part III scores in *LRRK2*-NMC compared to HC [32, 33•]. In a few studies using sensory-based gait analysis, the G2019S mutation was associated with increased arm swing asymmetry and variability during dual tasking or fast walking in cohorts of either NMC or PD patients [34, 35]. However, since these abnormalities were found in variant carriers, regardless of disease status, they could simply mirror the presence of *LRRK2*-G2019S, and not represent a true prodromal sign suggesting phenoconversion [34]. A subsequent study, conducted on a small cohort of individuals (27 *LRRK2*-G2019S-NMC and 36 HC), also found some differences in step time on the non-dominant side during fast-walking in NMC vs. controls and a negative correlation with dopamine transporter (DAT) uptake [36].

That said, are only *LRRK2*-G2019S-NMC the ones showing motor abnormalities, or is this a shared feature with other *LRRK2* variants-NMC? These questions have been addressed by a few studies with discordant results. One report suggested a variant-specific phenotype, with G2019S associated with pure motor symptoms and R1441G associated with non-motor alterations such as dysautonomia, depression, and sleep disorders [32]. Conversely, other studies detected increased autonomic dysfunction in *LRRK2*-G2019S-NMC, not accompanied by other prodromal symptoms [33•, 37]. Two longitudinal studies have focused on PD risk in *LRRK2*-G2019S or G2385R carriers. In a study conducted on healthy first-degree relatives of Ashkenazi Jewish *LRRK2*-G2019S-PD patients (120 G2019S carriers, 111 G2019S non-carriers), over 5 years, 4.3% of the individuals were diagnosed with PD, and all were G2019S carriers. Prodromal markers such as higher UPDRS-III scores, somnolence, constipation, and erectile dysfunction, were statistically more frequent in newly diagnosed PD cases [38••]. In another 10-year longitudinal study conducted on Chinese communities composed by carriers of *LRRK2*-G2385R variant (329 subjects) or non-carriers (345 subjects), an increased PD risk for *LRRK2*-G2385R-NMC above 50 years of age (7.9%) compared with non-carriers (2.6%) was detected; however, no differences in motor or non-motor symptoms were observed between the two groups [39].

In conclusion, at present, it remains unclear whether there is a variant-specific profile for *LRRK2*-NMC variants. Subtle motor changes seem to precede manifest motor onset at least in *LRRK2*-G2019S-NMC, and therefore, we recommend including sensory-based gait analysis as part of screening evaluation of healthy carriers.

## Genetic Markers Beyond *GBA1 *and *LRRK2*

Whether other genetic factors might act as modifiers of disease penetrance among carriers of *GBA1* variants has been investigated by recent large genome-wide association studies. Interestingly, Straniero and colleagues found that deleterious variants in lysosomal genes can modulate the risk of *GBA1*-PD, in particular a second variant in *GBA1* [40]. In another study, Blauwendraat and colleagues identified that variants near *SNCA* and *CTSB* contribute to increased PD risk and decreased age at onset, but the contribution of common genetic variants was small overall [41]. The occurrence of additional co-segregating rare variants in lysosomal genes was also found in families segregating *GBA1* variants [42].

Similarly, for *LRRK2* carriers, it has been suggested that non-*LRRK2* genetic factors may influence PD penetrance [43]. Polygenic risk score (PRS), for instance, was associated with higher penetrance of PD in large cohorts of *LRRK2*-G2019S carriers [44, 45], and the combination of MDS Research Criteria for Prodromal PD with PRS has been proposed for *LRRK2*-NMC to identify subjects at higher risk [3].

## Environmental Markers

In recent years, it has become evident that specific environmental factors can play an important role as modulators of PD penetrance.

Non-steroid anti-inflammatory drugs (NSAIDs) exposure has been proposed as marker of PD resistance [46]. A retrospective case–control study found that regular use (defined as two or more pills per week for 6 months or more) of NSAIDs (both aspirin and ibuprofen) may lower the risk for PD with OR of 0.34 among *LRRK2* variant carriers, including pathogenic (such as G2019S) and risk variants [47••]. Different hypotheses have been proposed to explain the inverse association between NSAIDs and *LRRK2*-PD. On the one hand, NSAIDs could play a role as modifier factors of inflammation secondary to *LRRK2* variants; on the other hand, NSAIDs intake could represent a marker of another determinant of reduced PD risk (for instance, caffeine intake or smoking) which is associated to NSAIDs use [48]. In support of the latter scenario, there is now increasing evidence suggesting a protective effect of caffeine intake and smoking towards PD development, particularly in *LRRK2* carriers. In fact, plasma metabolomic analysis showed that PD patients (70 sPD, 118 *LRRK2*-PD) had lower levels of plasma caffeine concentration compared with healthy individuals (65 HC, 115 *LRRK2*-NMC), more so in *LRRK2*-positive (by 76%) compared with *LRRK2*-negative carriers (by 31%), with significant interaction between *LRRK2* and PD status [49•]. Similar results were also found for caffeine demethylation metabolites (paraxanthine, theophylline, 1-methylxanthine) and trigonelline, a nonxanthine marker of coffee consumption [49•]. In this study, *LRRK2*-PD patients also reported reduced dietary caffeine intake compared with asymptomatic *LRRK2*-NMC [49•]. Similarly, two recent studies conducted on *LRRK2*-G2019S-PD from different geographical areas (Tunisia: 142 *LRRK2*-G2019S-PD, 200 sPD, 57 *LRRK2*-G2019S-NMC; Israel: 65 *LRRK2*-G2019S-PD, 100 sPD) showed an association between older PD age at onset and tobacco use and coffee and tea consumption [50, 51], suggesting a protective effect of these factors.

Overall, this evidence suggests that questions related to lifestyle habits (e.g., NSAIDs use, caffeine intake, and smoking) should become part of the initial screening of *LRRK2*-NMC, eventually followed by more refined analyses such as metabolomics, to further stratify individuals. Future studies should consider evaluating whether the association with these environmental factors is specific for *LRRK2* or they can play a role in other genetically stratified cohorts (e.g., *GBA1*-NMC).

## Biochemical Markers

To increase our ability to detect high-risk individuals, clustering clinical and historical data with biochemical markers represents a reasonable option [3].

### Alfa-Synuclein.

Levels of α-synuclein in plasma or peripheral blood mononuclear cells (PMBCs) have been found to be increased in *GBA1*-PD patients compared to sPD [52, 53]. Recently, Emelyanov et al. showed significantly increased CD45 + α-synuclein levels in a group of 12 *GBA1*-NMC when compared with HC, sPD, and *GBA1*-PD [54]. Despite the small sample size, a spectrum of increased α-synuclein levels was observed from *GBA1*-PD, to sPD, HC, and *GBA1*-NMC, suggesting that either this increase represents a compensatory mechanism that *GBA1*-NMC exert, or an early, pathological marker that can decompensate later.

Evaluation of α-synuclein levels within *LRRK2* carriers is more controversial. This may reflect the different neuropathological substrates associated with distinct *LRRK2* variants, with G2019S showing Lewy body pathology in 64% of cases and R1441G only in a few [55, 56]. In a former study, CSF total α-synuclein levels were found similar in *LRRK2*-NMC, *LRRK2*-PD, and HC but higher than in sPD [57]. Conversely, a more recent study showed lower levels of CSF total α-synuclein and increased levels of oligomeric α-synuclein in *LRRK2*-NMC than HC, but similar levels compared with PD patients with and without *LRRK2* variants [58]. The combination of total-α-synuclein, oligomeric-α-synuclein, and TNF-α could discriminate *LRRK2*-NMC from HC with an area under the curve of 0.843 [58]. Evaluation of CSF α-synuclein aggregates through real-time quaking-induced conversion (RT-QuIC) has also been proposed as a marker of possible phenoconversion in *LRRK2* carriers. In one study, three of 16 *LRRK2*-G2019S-NMC (18.8%) were positive for RT-QuIC reaction; none of them developed manifest signs of PD during a 3-year follow-up; however, this subgroup tended to present altered DAT SPECT and fit the criteria for prodromal PD more frequently than NMC without positive RT-QuIC response [59]. Although the discriminant power of CSF α-synuclein measurements is promising, lumbar puncture is an invasive procedure and therefore not applicable as a large-scale screening tool.

### Inflammation.

Due to highly expressed levels of *LRRK2* protein in peripheral immune cells, in particular neutrophil and classical monocytes, and its recognized central role in several immune and infection processes [60], the peripheral and central inflammatory profiles have been evaluated not only in *LRRK2*-NMC but also in *GBA1*-NMC, as potential factors modulating disease risk.

Initial studies evaluating *LRRK2*-G2019S-NMC, found higher levels of serum (not CSF) cytokines, especially IL-1β, when compared with HC. Interestingly, a subset of G2019S carriers displayed IL-1β levels similar to people with manifest PD, but these levels did not correlate with any early prodromal PD symptom [61]. A subsequent study comparing large cohorts of *LRRK2*-G2019S and *GBA1* variant carriers vs. non-carriers (either with or without PD) did not find any difference in serum or CSF cytokine levels between groups [26]. A separate study evaluating *GBA1*-NMC also did not show any difference in plasma cytokines levels in these individuals compared with HC [27]. The interpretation of these inconsistent results is challenging. It may be that for *LRRK2*, a peripheral, initial inflammation process is present at least for a subset of high-risk individuals, without any clear prodromal symptoms associated. For *LRRK2*-NMC, longitudinal studies evaluating peripheral cytokines as predictive markers might be worth pursuing, whereas, at this stage, invasive CSF analysis does not seem to be informative in the preclinical phase.

Blood urate, an endogenous antioxidant and end product of purine metabolism, is another anti-inflammatory factor which is considered a marker of resistance to PD [62], especially in the context of *LRRK2*-PD. A cross-sectional study evaluating *LRRK2*-PD vs. *LRRK2*-NMC showed that *LRRK2*-NMC had higher urate levels compared with *LRRK2*-PD [63]. These results were validated across three independent case–control datasets, and odds of developing PD were reduced by approximately 50% (OR = 0.48) among *LRRK2* carriers for each 2 mg/dl increment in blood urate concentration [63]. Although a recent randomized controlled trial failed to show any benefit from use of urate-elevating inosine treatment in early PD [64], urate measurement in *LRRK2*-NMC remains a promising prognostic marker of resistance/susceptibility to PD development, and application of urate-modifying treatments should be considered in early *LRRK2*-PD cases. Moreover, since women show reduced levels of plasma urate compared with men [65], urate levels could contribute to the similar penetrance of PD in women and men carrying a *LRRK2* variant [66], thus making it an important test to perform within *LRRK2*-NMC for prognostic purposes.

### Lipid Metabolism.

Various markers linked to lipid metabolism have recently become the focus of many groups working on *GBA1*-PD. First, the enzymatic activity of the lysosomal enzyme gene product glucocerebrosidase (GCase) was evaluated as potential marker of PD conversion. Although a stepwise increase in GCase activity was noticed between severe, mild, risk variant *GBA1*-NMC, and HC, no difference was detected between mild *GBA1*-PD and mild *GBA1*-NMC or between severe *GBA1*-PD and severe *GBA1*-NMC, indicating that GCase activity reflects genotype status but cannot be used as prognostic biomarker for PD conversion [28]. Second, one of the sphingolipids accumulating in GD, glucosylsphingosine (GluSph), was compared in 20 subjects with and without PD, carrying or not the mild N370S *GBA1* variant. Plasma GluSph levels were higher in the *GBA1* groups, regardless of PD status; other lipids such as the GCase substrate glucosylceramide, or GCase product ceramide, did not show any difference across groups [67].

Significant alterations in sphingolipids and glycerolipids profile were recently shown in CSF of PD patients and *LRRK2*-NMC studied by untargeted high-performance liquid chromatography-tandem mass spectrometry; however, there was no correlation between CSF and serum lipidomes [68]. Lipidomic profile could therefore find its niche as a marker in clinical trials targeting *LRRK2*, but its applicability as prognostic marker of phenoconversion is unsure.

### Routine Blood Markers.

In a study evaluating general biochemical markers (full blood count, renal function, C-reactive protein, and vitamin D) in *GBA1*-NMC, *LRRK2*-NMC, and HC, a positive association between sub-clinical renal impairment and higher likelihood for prodromal PD was detected among all non-manifesting subjects. Moreover, higher probability for PD was found to be associated with lower levels of vitamin D and hemoglobin in *LRRK2*-NMC, only [25]. The same group also found that *LRRK2*-NMC had higher triglyceride levels compared to *GBA1*-NMC and HC; irrespective of genotype, those NMC with probability rates for prodromal PD above 50% had higher frequencies of hypertriglyceridemia and prediabetes [69]. Overall, these findings suggest that these measurements might be useful to identify subjects at higher risk of PD, but they are not specific for genetically defined cohorts.

### Pathogenic Mechanisms.

Use of analyses such as transcriptomics or proteomics can provide insight into the pathogenic mechanisms implicated in PD development in genetically stratified cohorts; however, their applicability in clinical setting is difficult. For instance, transcriptomic profile of monocytes in a cohort of *GBA1*-PD, sPD, *GBA1*-NMC, and HC, did not find any specific gene target or biological process related to *GBA1* signature, but gene-based outlier analysis in *GBA1*-NMC showed involvement of mitochondrial function [70]. When the authors performed the same analysis in an independent and larger cohort of whole blood samples, they only marginally replicated the results suggesting cell-specificity [70]. Another recent study applied unbiased mass-spectrometry phospho-proteomic study in PBMCs to a cohort of *LRRK2*-G2019S and *LRRK2*-R1441G carriers (with and without PD). The authors detected specific protein differences between *LRRK2*-G2019S-PD, *LRRK2*-G2019S-NMC, and HC, with the largest protein phosphorylation differences between *LRRK2*-G2019S-PD and *LRRK2*-G2019S-NMC, suggesting the presence of specific phosphorylation events associated with changes from asymptomatic to manifest PD [71].

Bis(monoacylglycerol) phosphate (BMP) isoforms, which contribute to the multivesicular/lamellar morphology of the endolysosomal network, were higher in urine of carriers of *LRRK2*-G2019S than in non-carriers, independent of PD status [72]. Only the 2,2′-di-18:1-BMP isoform was marginally higher in *LRRK2*-G2019S-PD when compared with *LRRK2*-G2019S-NMC; therefore, whether these markers may be useful to predict PD conversion is unclear [72].

## Neuroimaging Markers

Conventional and advanced imaging techniques constitute another interesting tool used to provide objective in vivo measurements of brain dysfunction, which may predate clinical manifestations of disease within genetically stratified cohorts [73]. For a comprehensive review on the topic, the reader is directed elsewhere [73]. Below, we will present the most recent neuroimaging studies evaluating *GBA1*-NMC and *LRRK2*-NMC, highlighting the successful/unsuccessful application of such imaging techniques as prediction markers.

### Structural and Functional MRI (fMRI)

No structural brain changes (subcortical volumes and cortical thickness) have been reported in *GBA1*-NMC and *LRRK2*-NMC compared with HC [74, 75]. Conversely, alterations in resting state fMRI in *GBA1*-NMC have been reported in a few studies. An increased resting state striato-cortical functional connectivity (FC) between left posterior putamen and left postcentral gyrus and hyper-connectivity between left caudate and right parietal operculum and right planum temporale were detected in a small group of *GBA1*-NMC compared with HC, suggesting a possible early impairment of sensory system in these individuals [74]. In another fMRI study, despite similar performances on the Stroop test, *GBA1*-NMC showed increased task-related activity in the right medial frontal gyrus and reduced task-related activity in the left lingual gyrus compared with *LRRK2*-NMC and HC and higher activation patterns in the incongruent task in the left medial frontal gyrus and bilateral precentral gyrus compared with HC. These findings were interpreted as a compensatory mechanism allowing adequate cognitive performance [76], and therefore, they could simply reflect *GBA1* status, and not be associated with increased risk of PD.

### 123-I Ioflupane DAT Imaging

Similar to fMRI, studies evaluating DAT striatal-binding ratios (SBRs) have found little and inconsistent alterations across studies. In a large cohort of *GBA1*- and *LRRK2*-NMC, only 3% of *GBA1*-NMC and 11% of *LRRK2*-NMC displayed DAT deficit. Interestingly, *GBA1*-NMC, but not *LRRK2*-NMC, showed increased DAT SBRs in the caudate, putamen, and striatum compared with HC, and this was interpreted as a compensatory mechanism [33•]. Conversely, in another study comparing iRBD, individuals with hyposmia, and *GBA1*-NMC and *LRRK2*-NMC, a lower mean SBR was observed in iRBD compared with hyposmia and NMC cohorts, and no differences were found between *GBA1*-NMC and *LRRK2*-NMC [77]. Using baseline and annual change rates in longitudinal data obtained using DAT SPECT from sPD, *LRRK2*-G2019S-PD, and *GBA1*-N370S-PD patients, Lee et al. tried to predict the temporal trajectory of putaminal dopaminergic dysfunction during the premotor phase. These authors suggested that the presence of dopaminergic degeneration in *GBA1*-PD was present 10 years before motor onset and that *GBA1*-PD patients had faster deterioration of dopaminergic function compared with both *LRRK2*-PD and sPD [78].

To increase the sensitivity of these imaging data at predicting PD, a recent study combined the evaluation of FC patterns and presynaptic striatal dopamine uptake DAT SPECT, in a group of 26 *GBA1*-NMC, 25 *LRRK2*-NMC, and 34 HC. Despite similar clinical features across groups, *LRRK2*-NMC showed reduced SBRs in the right putamen compared with HC, while no significant differences in SBRs were detected between *GBA1*-NMC and HC [79]. *LRRK2*-NMC also showed higher right putamen FC compared with *GBA1*-NMC. Within the *LRRK2*-NMC, higher striatal FC was associated with increased risk for PD. Within 3 years of follow-up, three individuals (2 *GBA1*-NMC and 1 *LRRK2*-NMC) converted to PD, and they were found to be 2 or 1 SD below their respective groups measured intra-striatal mean FC level [79].

### Miscellaneous

Microglial activation has been measured in vivo using ^11^C-(R)-PK11195 positron emission tomography (PET) imaging on a small cohort of *GBA1* variant carriers (including GD and heterozygous *GBA1*-NMC), showing that ^11^C-(R)-PK11195-binding potential was increased in the substantia nigra of *GBA1* variant carriers compared with HC and correlated with hyposmia [80]. Mean striatal ^18^F-fluorodopa (F-DOPA) PET was similar to HC, and therefore, it was proposed that *GBA1* variant carriers might show increased microglial activity in brain regions susceptible to neuropathological changes, either as a result of cytotoxic or neuroprotective process [80]. F-DOPA PET imaging was also investigated in a longitudinal study evaluating *GBA1*-NMC (both heterozygous and homozygous). No significant difference in striatal tracer uptake was observed compared with HC after a maximum of 9-year-follow-up [81].

Recent studies using [^18^F]-fluorodeoxyglucose (FDG) PET demonstrated altered brain metabolism in *GBA1*-PD compared with *LRRK2*-PD and sPD, underlying a more aggressive disease in *GBA1*-PD patients [82, 83]. Although no data are available to date in NMC, [^18^F]-FDG PET may be a promising tool to detect impaired brain metabolism in genetically at-risk individuals as recently shown in other PD at-risk groups like iRBD [84].

Overall, these neuroimaging data suggest that differential neuroimaging patterns can be observed among genetically at-risk individuals and that combined DAT and FC assessments might increase the ability to detect high-risk subjects [79]. Other imaging techniques such as fMRI or F-DOPA PET might not be sensitive markers to predict PD conversion in these populations.

## Conclusions

**Fig. 1:**
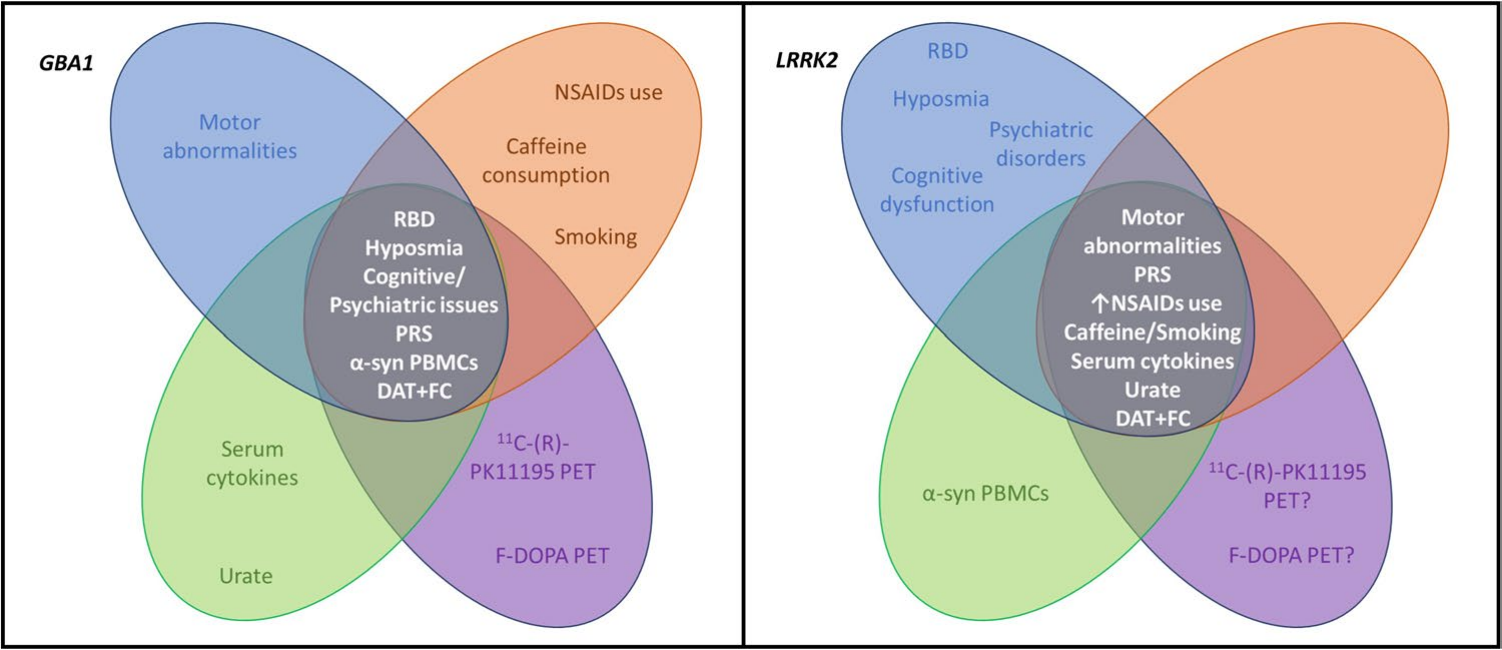
Summary of clinical (blue), genetic/environmental (orange), biochemical (green), and imaging (purple) markers that could be more relevant to define higher risk of PD conversion in *GBA1*-NMC and *LRRK2*-NMC. FC, functional connectivity; PBMCs, peripheral blood mononuclear cells; PRS, polygenic risk score

Over the last few decades, our understanding of clinical and pathological differences in *GBA1*-PD and *LRRK2*-PD has greatly increased; however, the issue of identifying those at greatest risk of conversion to PD remains beyond our capabilities. Review of all the potential prodromal markers of PD phenoconversion in *GBA1* and *LRRK2* variant carriers leads us to the conclusion that at present no single clinical, biochemical, or imaging feature, or combination thereof can either identify those most likely to convert or when. This represents an important challenge as the ability to do so would enable those individuals at risk to be offered preventative therapy that should ever become available. In Fig. 1, we suggest which markers might be relevant, or not, for *GBA1* and *LRRK2* variant carriers and should therefore be assessed in individuals carrying these variants. To validate whether these proposed preclinical/prodromal markers can reliably distinguish those individuals that are truly premotor PD, we need longitudinal studies of large preclinical cohorts, using a combination of clinical, historical, and genetic information, together with biochemical/imaging data, and artificial intelligence approaches to create trajectory models. These studies will pave the way for personalized therapeutic approaches.
